# Towards defining biomarkers to evaluate concussions using virtual reality and a moving platform (BioVRSea)

**DOI:** 10.1038/s41598-022-12822-0

**Published:** 2022-05-30

**Authors:** Deborah Jacob, Ingunn S. Unnsteinsdóttir Kristensen, Romain Aubonnet, Marco Recenti, Leandro Donisi, Carlo Ricciardi, Halldór Á. R. Svansson, Sólveig Agnarsdóttir, Andrea Colacino, María K. Jónsdóttir, Hafrún Kristjánsdóttir, Helga Á. Sigurjónsdóttir, Mario Cesarelli, Lára Ósk Eggertsdóttir Claessen, Mahmoud Hassan, Hannes Petersen, Paolo Gargiulo

**Affiliations:** 1 Institute of Biomedical and Neural Engineering, Reykjavik University, Reykjavik, Iceland; 2 Department of Psychology, School of Social Sciences, Reykjavik University, Reykjavik, Iceland; 3 Department of Chemical, Materials and Production Engineering, University of Naples Federico II, Naples, Italy; 4 Department of Electrical Engineering and Information Technology, University of Naples Federico II, Naples, Italy; 5 Department of Computer Engineering, Electrical and Applied Mathematics, University of Salerno, Salerno, Italy; 6 Landspitali National University Hospital of Iceland, Reykjavik, Iceland; 7 Physical Activity, Physical Education, Sport and Health (PAPESH) Research Centre, Sports Science Department, School of Social Sciences, Reykjavik University, Reykjavik, Iceland; 8 Faculty of Medicine, School of Health Sciences, University of Iceland, Reykjavik, Iceland; 9 Department of Information Technology and Electrical Engineering, University of Naples, Naples, Italy; 10 MINDig, 35000 Rennes, France; 11 Department of Anatomy, Faculty of Medicine, School of Health Sciences, University of Iceland, Reykjavik, Iceland; 12 Akureyri Hospital, Akureyri, Iceland; 13 Department of Science, Landspitali, National University Hospital of Iceland, Reykjavik, Iceland

**Keywords:** Biomedical engineering, Brain injuries, Biomarkers, Diagnostic markers

## Abstract

Current diagnosis of concussion relies on self-reported symptoms and medical records rather than objective biomarkers. This work uses a novel measurement setup called BioVRSea to quantify concussion status. The paradigm is based on brain and muscle signals (EEG, EMG), heart rate and center of pressure (CoP) measurements during a postural control task triggered by a moving platform and a virtual reality environment. Measurements were performed on 54 professional athletes who self-reported their history of concussion or non-concussion. Both groups completed a concussion symptom scale (SCAT5) before the measurement. We analyzed biosignals and CoP parameters before and after the platform movements, to compare the net response of individual postural control. The results showed that BioVRSea discriminated between the concussion and non-concussion groups. Particularly, EEG power spectral density in delta and theta bands showed significant changes in the concussion group and right soleus median frequency from the EMG signal differentiated concussed individuals with balance problems from the other groups. Anterior–posterior CoP frequency-based parameters discriminated concussed individuals with balance problems. Finally, we used machine learning to classify concussion and non-concussion, demonstrating that combining SCAT5 and BioVRSea parameters gives an accuracy up to 95.5%. This study is a step towards quantitative assessment of concussion.

## Introduction

A concussion, or mild traumatic brain injury (mTBI), is a short-lived functional neurological impairment caused by a blow to the head or by a force transmitted to the head^1^. Participation in sports is a risk factor for sustaining multiple concussions^6^, with some sports presenting a greater risk than others^7,8^. The possible acute symptoms of concussion can include headaches, emotionality, loss of consciousness, amnesia, problems with balance, and sleep/wake disturbance^4,5^. Although most cases of concussions resolve spontaneously, they can have persistent psychological, physical, and cognitive complications and protracted recovery times^1–3,7,9–13^.

There is currently no objective way to diagnose a concussion^1^, nor is there a precise, universal concussion definition^14^. When diagnosing concussions, medical professionals rely on clinical assessment, which can be problematic as symptoms are non-specific^4^ and could relate to other illnesses, mental or physical. In research, concussion assessments are usually carried out through questionnaires built around the most recent consensus on the definition of concussion^4^. The Concussion Assessment Tool, fifth edition (SCAT5), has been useful when assessing symptoms after an incident and tracking recovery^4^. Although it is easily administered, it should not be used on its own for diagnosis, not unlike other similar concussion assessment scales^26,111–113^. Medical records and clinical interviews are sometimes thought of as the gold standard in concussion research^15^, inevitably resulting in a loss of accuracy as many who suffer from a concussion do not seek medical assistance^16^.

The pathology of concussions has been described as puzzling, and it is unclear how changes in neuronal function affect the development of concussion symptoms^3^. Even though a concussion can be diagnosed in the absence of structural damage, structural damage has been found after a concussion ^4^ possibly contributing to prolonged symptoms^27^. Although not routine in clinical practice, neuroimaging might help us understand concussion symptoms and how they relate to functional changes in the brain (for a review of functional MRI techniques in concussion studies, see^27^). Electrophysiological assessments of concussions also show promise. Electroencephalography (EEG) serves as a non-invasive method to measure electrical activity, providing insight into brain activity connected to concussion pathology^3^ and has been used to identify functional changes in the brain following a concussion^28–33^. EEG is more manageable and inexpensive than many other brain imaging techniques^32^ and although still needing special training, it is more accessible for researchers and clinicians. In the first known study to assess steady-state visual-evoked potentials (SSVEP) in concussed athletes, researchers found a difference in the SSVEP compared to healthy athletes^34^, despite only using visual stimuli. Further, reduced brain network activation, measured by an EEG, has been related to post-traumatic migraine (PTM) in concussion patients, possibly indicating symptom severity or persistency as PTM can correlate with increased severity and prolonged recovery^35^. Concussion history has also been related to a change in theta and alpha activity^36,114–116^. However, because methodologies vary between studies it is challenging to establish EEG markers as return-to-play guidelines^29,32,41^. More research into the relationship between EEG and subjective concussion symptoms is needed. For EEG to be used to establish return-to-play guidelines it is important to include assessments using more than visual stimuli. More demanding actions, including physical movement, should be assessed in conjunction with EEG to be closer to real-world situations likely to induce concussion symptoms. Supporting this are findings that demonstrate a relationship between concussion and altered postural control^52,53^. When paired with a postural task, EEG signals have indicated that concussion sequelae could be found months after an injury^36^. One study even found a change in EEG measured 30 years after a concussion^47^. Further, EEG measures have been found to predict concussion with reasonable accuracy when using machine learning methods^38^. These results indicate that EEG parameters are possible biomarkers for concussion.

Among the most common post-concussive symptoms are dizziness and balance problems^17^. The objective assessment of postural control is, therefore, a natural candidate for investigation among those who have suffered a concussion as postural control^117,118^ depends on inputs from visual and vestibular systems and the somatosensory system^23^, commonly affected by a concussion. Centre of pressure (CoP) has been assessed in the maintenance of upright stance and is an important measure of postural stability^119^ and as a possible indicator of a concussive injury^118,120^. While postural control and the connection to concussion are often assessed by having participants stand on a pressure plate, more sophisticated measures and analysis should be incorporated^121^. In Degani et al.^44^, participants with a history of mTBI presented a larger, slower, and more random body oscillation than controls when body CoP displacement was assessed by having participants stand on a force plate while performing a simple task. CoP displacement has also been shown to correlate with EEG measures in an mTBI group, up to one year after injury^54^. Postural control assessed with the sensory organization test (SOT) following a mTBI^23^ showed that those who had previously experienced a concussion displayed different postural control dynamics compared to a control group, months and up to several years after the initial injury. This relationship between concussive impacts and balance problems has been demonstrated in many studies, with targeted quantitative methods focusing on sensorimotor and neural components suggested as the next step for better understanding^122^. Given this connection between concussion and postural control, electromyographic (EMG) recordings, although not commonly used when assessing symptoms after concussion^39^, could be used to assess possible biomarkers for concussion, as they can record muscular activity necessary to maintain postural stability ^42^. An abnormality has been found in EMG signals represented as a pause after a motor evoked potential, positively correlated with injury severity among athletes with a concussion^40^. The tibialis anterior (TA) plays a key role in stability control^51^, and EMG recordings from the TA have been used in postural control studies^25,43^. For a more detailed overview of EEG and EMG in concussion studies, see Table 1.

**Table 1:** summarizes the author´s literature review in quantitative assessment of concussion using neurophysiological signals.

Modality	Condition	Results	Publication details
**EEG Configuration**			
Various	Resting state	Literature review of rs-EEG reporting heterogeneous results when comparing concussion vs non-concussion groups due to large methodological differences in power, coherence, and continuous EEG studies	^29^
Not stated	Resting State	Short literature review detailing spectral, amplitude, coherence, and homologous EEG studies in acute, subacute, and chronic mTBI versus normal groups. No concrete marker for mTBI found in the literature using quantitative EEG methods	^30^
9 electrodes at F3, F4, Fz, C3, C4, Cz, P3, P4 and POZ	Cognitive task, eyes open, eyes closed	Concussed group exhibited increase in delta and theta bands compared to controls	^31^
		Lower frontal theta to gamma ratio in concussion group	^33^
9 electrodes at F3, F4, Fz, C3, C4, Cz, P3, P4 and POZ	Motor Task—target capture, perception span and hand reaction time	Concussion group displays decreased EEG power in all bandwidths, especially in standing postures accompanied by sustained postural instability in eyes closed	^36^
19 electrodes placed according to 10–20 system	Eyes open, eyes closed and both seated and static standing		
14 electrodes	Steady state visually evoked potential	Concussion group had lower signal to noise ratio compared to a previously measured baseline during acute concussion stage (< 72 h after concussion)	^34^
128 electrode cap	Resting state	Decreased brain network activation in those with posttraumatic migraine after suffering concussion	^35^
40-channel EEG		Delayed and attenuated P3a P3b responses in the concussion group	^37^
**EMG**			
-Vestibular Evoked Myopotentials (VEMPs)	Auditory Evoked Potential	Reduced VEMPs measured in chronic dizziness/postural control difficulty group following blast exposure	^42^
-Surface EMG (sEMG)	Standing postural control	Better proprioceptive acuity predicts more co-contraction of the tibialis anterior and gastrocnemius lateral in older adults despite similar levels of CoP displacement between them and a younger control group	^44^

Blood flow and heart rate (HR) present other objective measures and have been found helpful in clinical settings when assessing individual differences in outcome after a concussion^45,46^. Changes in HR variability in concussed athletes have been shown weeks or months after an incident^47^, showing the potential of the autonomic nervous system (ANS) to function as a marker of concussion^46,48^. However, results from HR studies are mixed, and it is recommended to use measures of HR variability only as a part of a multi-faceted approach^46^, considering sex and age^49^. A better understanding of how HR measures relate to concussions is needed as they could offer a cost-effective and non-invasive way to track concussion recovery^46^.

Due to the complex etiology of concussive symptoms, a multi-faceted approach to concussion assessment and treatment is essential^24^, including multiple concussion measures and techniques. As a part of a multi-faceted approach, virtual reality (VR) offers a novel way to evaluate and manipulate postural control and cortical activity^54^. VR gives an option to assess responses in a secure setting while exposing participants to a visually and physically demanding task. In recent years, VR has been recognized as a valuable tool, both as a stimulus and as a measurement tool offering new ways to study psychological and behavioral factors related to health^123^, even showing promise in detecting and treating Alzheimer´s dementia^124^. In one study, EMG measures were acquired in a VR environment among high-risk concussion participants^55^. The experiment was a preliminary study to verify data in a VR environment. Results showed promise, although more research is needed. A 2020 study by Rao et al. ^56^ used VR, a moving platform, lower limb accelerometry, and EMG to detect subtle differences in balance between an mTBI group and a normal control group. Using these biometric measurements, walking and standing perturbation tests discriminated between the two groups. Results indicated that clinical assessment of concussion could be missing an important component, in this case, gait. By including movement, the assessment could be improved and with the addition of VR, the possibilities in manipulating and changing research settings in real-time greatly refined. The study also shows that a highly instrumented, multi-modal VR environment used in the performance of a demanding task can add important information that is not available in the clinical environment when assessing a concussion.

When using a multi-faceted approach, it is essential to carefully consider how the different data values should be processed to represent the construct being best assessed. The use of Machine Learning (ML) can be found throughout various scientific fields, offering a way to classify large datasets by using learning algorithms^97,125^. ML is used in multiple biomedical areas, including concussion research, and its efficacy has been positively demonstrated in recent scientific literature. Both supervised and unsupervised ML models can be used to study concussions^98,99^. An unsupervised clustering approach has been used^100^ to identify and characterize distinct concussion subtypes, while Visscher et al.^101^ used a self-organizing map to divide subjects with and without vestibular disorders after a concussive event. The supervised approach ranges from deep learning for concussion prediction on professional athletes^102^ to fuzzy trees algorithms to predict post-concussion symptom recovery^103^. Of relevance is the Castellanos et al. study from 2021^104^ with the use of a large but unbalanced dataset with the linear ML approach of the Support Vector Machine which allowed the prediction of sport-related concussion risk with significant accuracy.

**Figure 1:**
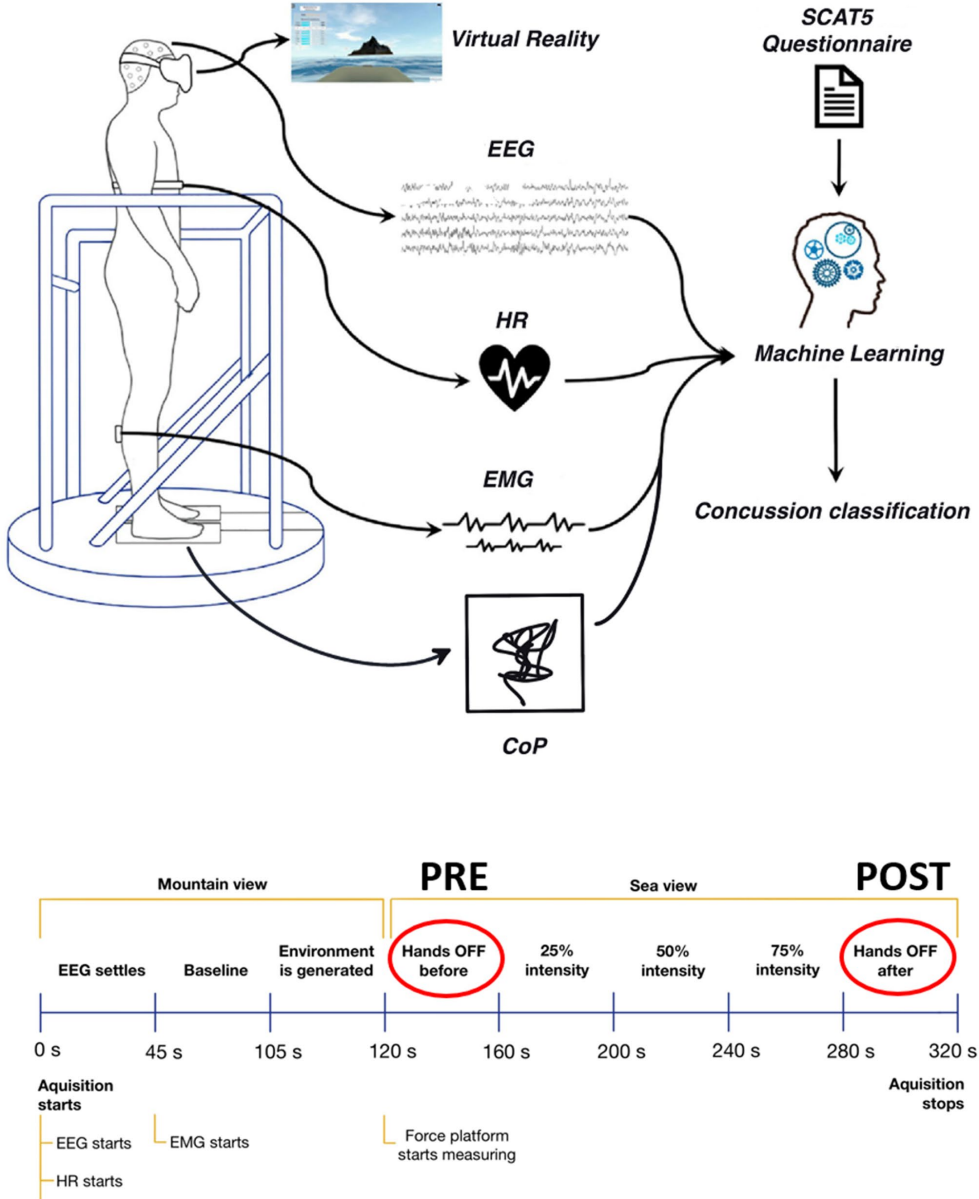
BioVRSea experimental Setup. BioVRSea is a novel VR-motion platform coupled system designed to mimic the sensation of being at sea in a small boat. Participants in this study stood on the platform while wearing VR goggles, EEG, and EMG measurement equipment while simultaneously having CoP measurements taken by two force platforms under each foot. The platform motion was coupled to the sea scene, as observed through the VR goggles. The protocol from the experiment included a baseline segment where the platform was not moving, and the participants stood with their hands by their side and viewed a VR scene of mountains. After two minutes the VR scene changed to that of a small boat at sea, the platform does not move for the first 40 s of the VR sea scene. The participant remains standing quietly with their hands by their side. This is the PRE part of the experiment. After 40 s, the participants are told to hold onto the safety bars for the following part of the experiment, where the platforms moves synchronously with the waves in the VR scene. There are three segments where the platform moves for 40 s at 25%, 50% and 75% of the maximum amplitude. The platform then stops moving and the participant is asked to remove their hands from the bar and stand quietly watching the VR sea scene for the final 40 s of the experiment. This is the POST phase.

This paper aimed to validate concussion/non-concussion classification and quantitatively assess different physiological responses during postural control tasks associated with concussion symptoms. The study was conducted on a homogenous cohort of female athletes with a background in sports with high impact contact. The athletes' self-reported concussion history was used to divide them in two groups: concussions and non-concussion. We first validated concussion history by asking them about concussion symptoms, using the SCAT5 questionnaire. Next, concussion groups were assessed in a novel measurement setup called BioVRSea (Fig. 1). Here, virtual reality and a synchronized moving platform were used to trigger a postural control reaction while measuring EEG, EMG, heart rate and center of pressure (CoP) parameters. This measurement allowed the assessment of physiological, neural and balance parameters during the simulation of a small boat at sea. The experiment was divided into five segments: in the first segment, the VR simulation was ON, but the platform was OFF (PRE). In the second segment, the VR simulation was ON, and the platform was ON at 25% of the maximal wave intensity. In the third segment, the VR simulation was ON, and the platform was ON at 50% of the maximal wave intensity. In the fourth segment, the simulation was ON, and the platform was ON at 75% of the maximal wave intensity. In the last segment, the simulation was ON, and the platform was OFF again (POST) (Fig. 1). We analyzed the results of POST minus PRE (POST–PRE) conditions for all measurements. We applied this paradigm to quantify the difference between concussion and non-concussion groups and concussion subgroups defined by different concussion symptoms. Finally, we computed the different measurements for each individual using machine learning techniques with the aim of classifying concussions based on self-reported and measured parameters. We hypothesized that (1) The Icelandic versions of the SCAT5 symptoms checklist, although not a diagnosis tool, can be used to differentiate between concussed and non-concussed athletes (2) Changes of CoP, heart rate, EMG and EEG data can quantitatively measure concussion and concussion symptoms, (3) machine learning techniques using SCAT5 and neurophysiological parameters can improve assessment of concussion.

## Results

Symptom or behavioral assessment with SCAT5 was performed prior to the instrumented VR experiment and is reported below, followed by the neurophysiological and classification results.

### Behavioral assessment

#### SCAT5 results

We used the SCAT5 to validate the self-reported concussion status, and to assess the changes in physiological conditions associated with our experiment. An independent t-test showed a significant difference between severity scores on the SCAT5 when comparing the concussed and the non-concussed athletes. Those with a history of concussion reported more severe concussion symptoms as assessed by the SCAT5 compared to those with no history of concussion (Table 2).

**Table 2:** Severity scores on the concussion scale (SCAT5) before VR acquisition.

	Athletes with no history of concussion			Athletes with a history of concussion			t-value
	N	M	SD	N	M	SD	
*SCAT5*							
Before	28	3.71	4.50	26	36.47	23.71	−7.18*

*p < 0.05.

**Figure 2:**
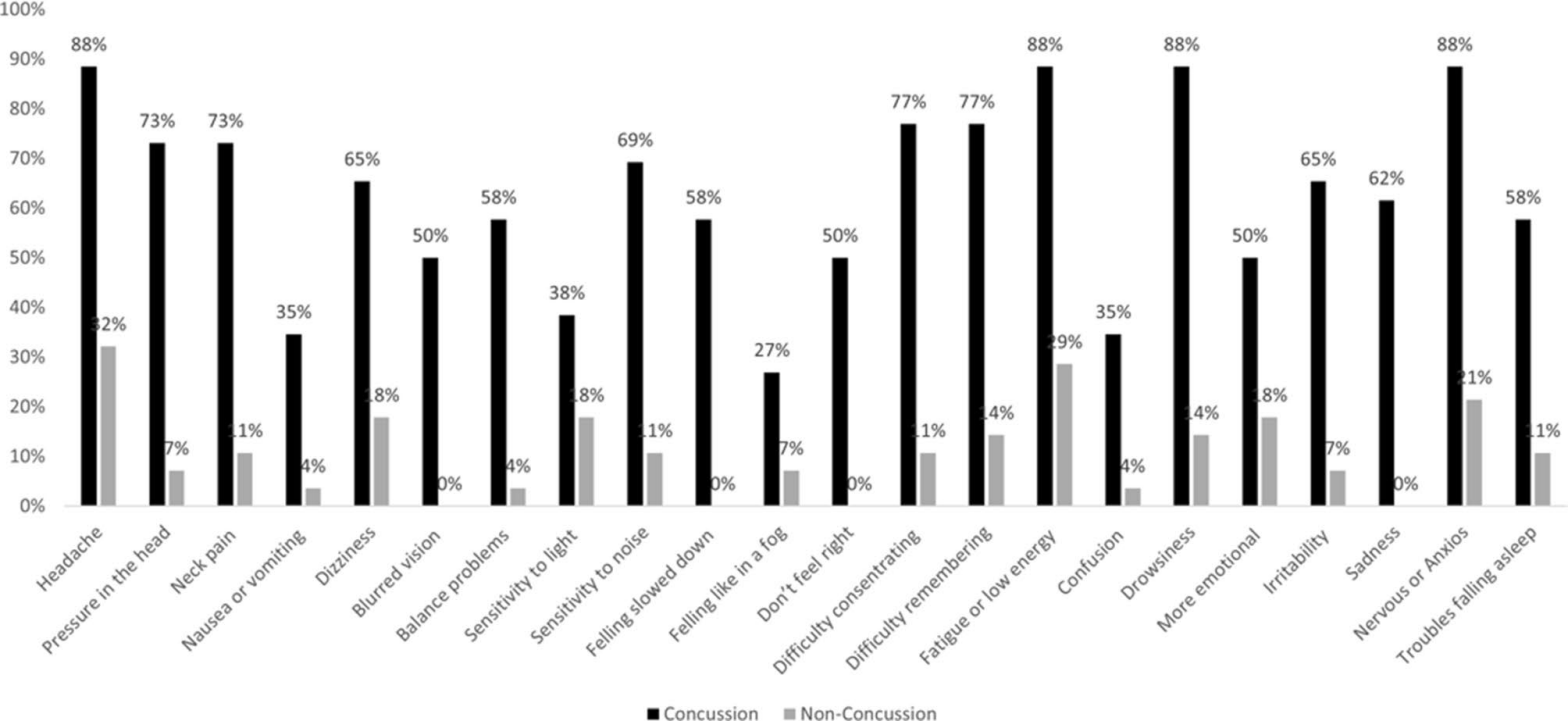
Symptoms (SCAT5) reported by all participants before and after VR acquisition. Overview of concussion symptoms reported by all participants before VR acquisition.

A closer look was taken at types of symptoms in the two groups. As seen in Fig. 2, 21% of participants with no concussion history reported feeling nervous and anxious before going into the VR environment compared to 88% of participants with a history of concussion (Fig. 2). The symptom most often reported among those without a concussion history was a headache, the only symptom above 30% (32%). Only one symptom was reported by under 30% of participants with concussion history, the symptom *feeling like in a fog*. The most reported symptoms in the concussion group were headache, fatigue/low energy, drowsiness and nervous/anxious.

#### Neurophysiological and balance measures

All CoP and neurophysiological parameters were studied as the difference of their values POST–PRE. In these two phases the subjects experienced the same environmental condition with the difference that in POST the subjects had to adapt to the induced movement provided from the previous phase where the platform was moving.

#### CoP analysis

We extracted several parameters from the stabilogram for evaluating the postural control response of the subjects before (PRE) and after (POST) the joined perturbation caused by the movement of the platform (25%, 50%, 75%) and the visual cues provided by the VR goggles. Six features from the displacement and velocity analysis showed significant differences (all *p* < 0.05) between the PRE and the POST stages for all groups.

- TOTEX—Total excursion.
- MDIST_AP—Mean distance from center in antero-posterior direction.
- MVELO—Mean velocity.
- ELLIPSE_MAIN_AXIS—The length of the main axis of 95% confidence ellipse.
- SD_AP—Standard Deviation in antero-posterior direction.
- POSTERO_MAGNITUDE—Posterior extreme point distance.

The concussion group exhibited larger changes from PRE to POST for all six features compared to the non-concussion group.

**Figure 3:**
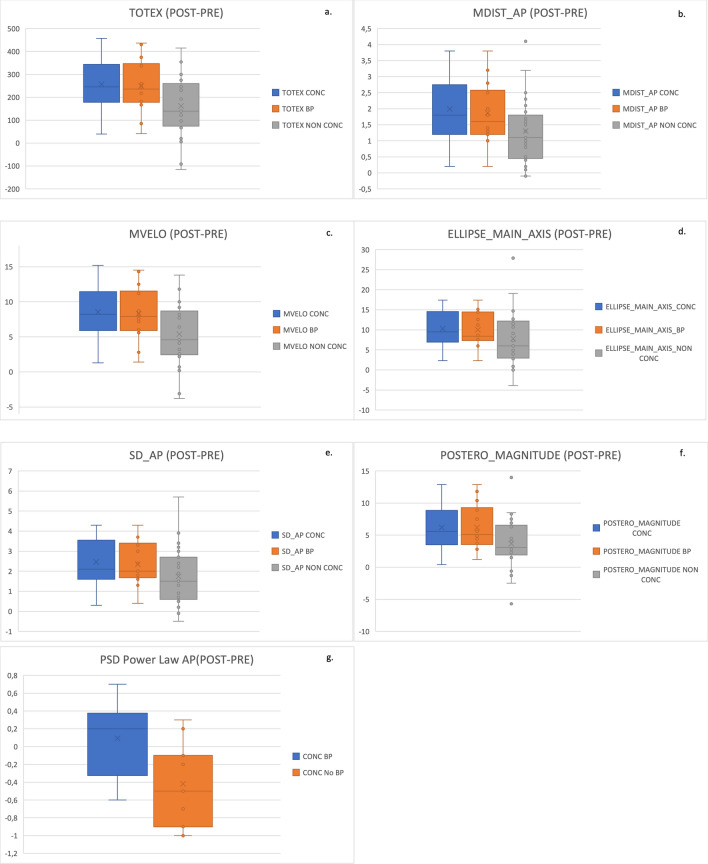
The most statistically significant features computed for the CoP parameters. All groups (Concussion (CONC—blue), Non-concussion (NON CONC—grey) and Balance Problems (BP—orange) showed a statistically significant change from the PRE to the POST stage for TOTEX (Total Excursion), MDIST_AP (Mean distance from centre in antero-posterior direction), MVELO (Mean velocity), ELLIPSE_MAIN_AXIS—The length of the main axis of 95% confidence ellipse, SD_AP (Standard Deviation in antero-posterior direction), POSTERO_MAGNITUDE (Posterior extreme point distance). One spectral feature PSD_EXP_AP—Power Spectral Density (PSD) Power Law is also shown.

One feature in the spectral analysis, PSD_EXP_AP – Power Spectral Density (PSD) Power Law Exponent for the AP signal showed statistical significance (p = 0.019) while comparing the concussion subgroup experiencing balance problems compared with concussion subgroup not experiencing balance problems. This can be seen in Fig. 3 g.

Calcualtion of features in both the anterior–posterior and medio-lateral directions were made, along with sample and multiscale entropy features, however they did not show any significant differences between PRE and POST, and are not reported on here.

### EEG analysis

**Figure 4:**
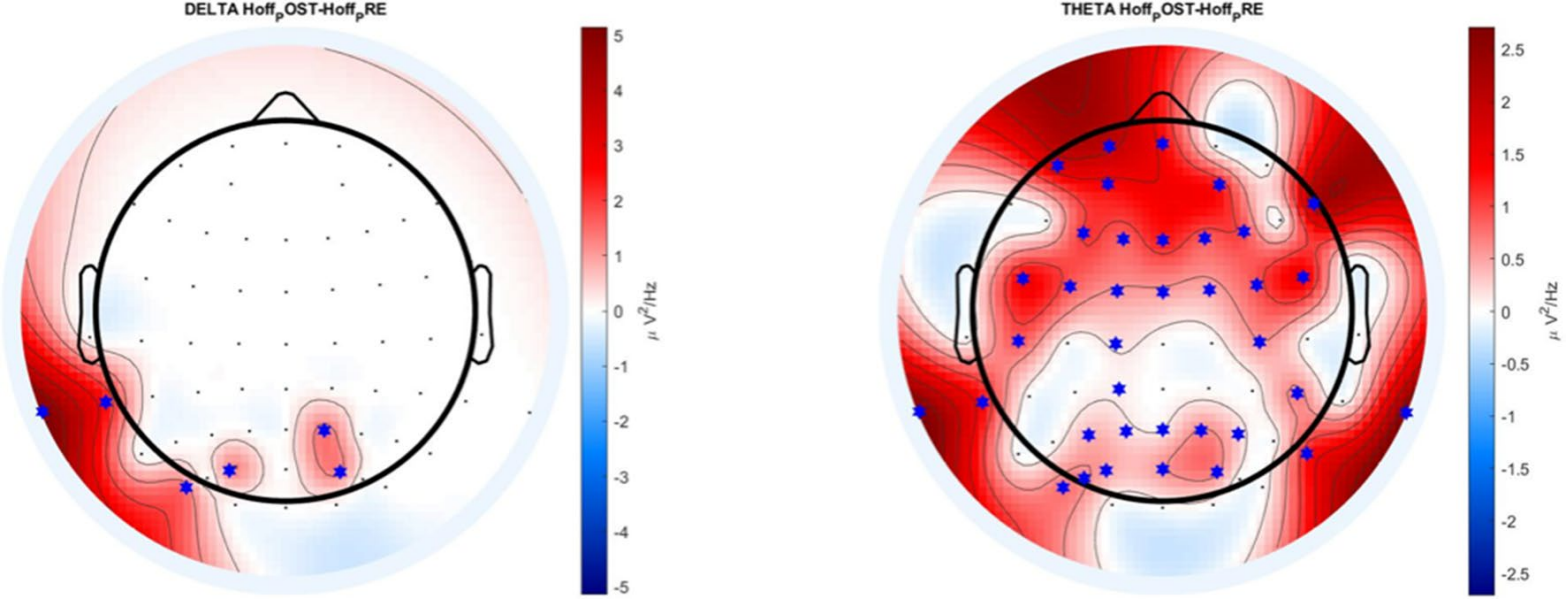
**A** topological plot for delta and theta frequency band of the concussion group. It displays the difference of power spectral density, between POST and PRE, only for the statistically significant electrodes (0.032 ≤ *p* ≤ 0.047, represented by a blue star in the figure). The delta band presented a few significant electrodes (6 out of 64) on the frontal and occipital cortex, whereas the theta band showed more distributed significant electrodes (36 out of 64) over the scalp.

Figure 4 shows the results from the EEG analysis performed in the frequency domain. The figure shows the difference POST–PRE for the concussion group for the delta and theta band, the only bands that were significantly different. The ‘*x*’ represents the electrodes for which we found significant differences for each band.

The difference was highlighted by an increase of power and significant evolution in the theta band mostly, and delta band for the concussion group (*p* = 0.038), the theta band showing significantly higher POST power than PRE. The theta band displayed an important number (37 out of 64) of significant electrodes (0.032 ≤ *p* ≤ 0.047) (*t*-test, corrected with false discovery rate (FDR), Benjamini–Hochberg method), in the frontal (18 out of 64 electrodes) and occipital (11 out of 64) cortex. The non-concussion group did not display any significant results for this experiment.

We then divided the concussion group into subgroups based on symptoms identified from the SCAT5 questionnaire. We analyzed the Difficulty concentration symptoms group (16 individuals), the Pressure in the head symptoms group (17 individuals), Fatigue and low energy group (19 individuals).

**Figure 5:**
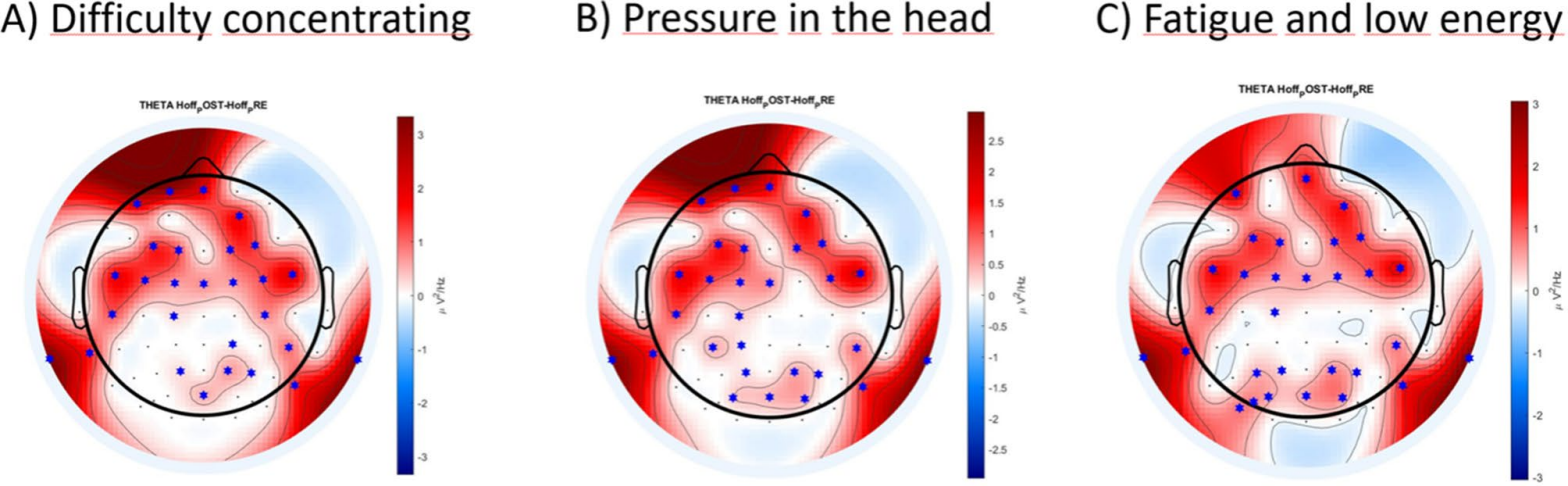
A topological plot of theta frequency band for three concussion symptoms subgroup. (**A**) Difficulty concentrating, (**B**) Pressure in the head, (**C**) Fatigue and low energy). It displays the difference of power spectral density, between POST and PRE, only for the statistically significant electrodes (0.008 ≤ *p* ≤ 0.049, represented by a blue star in the figure). Each subgroup presented significant electrodes on the frontal lobe (16 significant electrodes for difficulty concentrating, 15 for pressure in the head, and 14 for fatigue and low energy) for all of them, as well as an increase in the occipital lobe for the Pressure in the head (8 significant electrodes) and Fatigue and low energy (10 significant electrodes) subgroup.

Figure 5 shows the results from the EEG analysis performed in the spectral domain. The figure shows the difference POST–PRE for the concussion subgroups, for the theta band, the only band that presented significance. Figure 5A shows the results for the Difficulty concentrating group, 5B for the Pressure in the head group, and 5C for the Fatigue and Low energy group.

Figure 5 reveals that the subgroups present significant differences (0.008 ≤ *p* ≤ 0.049) for the theta band, with a global increase mostly in the frontocentral cortex (16 significant electrodes for difficulty concentrating, 15 for pressure in the head, and 14 for fatigue and low energy) for all of them, as well as an increase in the occipital lobe for the Pressure in the head (8 significant electrodes) and Fatigue and low energy (10 significant electrodes) subgroup.

Other symptoms groups were analyzed, such as headache (20 individuals), dizziness (14 individuals), balance problem (13 individuals) and more emotional group (11 individuals). These subgroups did not present any significant findings; therefore, their results will not be displayed.

Table 3 summarizes the significant electrodes (0.008 ≤ *p* ≤ 0.049) of Delta and Theta band, for the concussion group and the three subgroups detailed above.

**Table 3:** – Significant electrodes of delta and theta band for the concussion group and subgroups.

	Significant electrode Delta	Significant electrodes Theta
Concussion group	M1 P2 PO3 PO4 TP7 PO7	Fp1 Fpz F3 Fz F4 F8 FC5 FC1 FC2 FC6 M1 C4 M2 CP1 CP6 P3 Pz P4 P8 Poz AF7 AF3 AF4 F1 F2 FC3 FCz FC4 C5 C1 P1 P2 PO5 PO3 PO4 TP7 PO7
Difficulty concentrating subgroup	–	Fp1 Fpz F3 F4 FC5 FC1 FC2 FC6 M1 C4 M2 CP2 CP6 P4 P8 Poz AF7 AF4 F1 F2 FC3 FCz FC4 C5 C1 P1 P2 TP7
Pressure in the head subgroup	–	Fp1 Fpz F3 F4 FC5 FC1 FC6 M1 M2 CP1 CP6 P4 P8 Poz AF7 AF4 F1 F2 FC3 FCz FC4 C5 C1 CP3 P1 P2 PO3 PO4 TP7
Fatigue and low energy subgroup	–	Fpz F3 F4 FC5 FC1 FC2 FC6 M1 M2 CP6 P3 P4 P8 Poz AF7 AF4 F1 F2 FC3 FCz FC4 C5 C1 P1 P2 PO3 PO4 TP7 PO7

### EMG analysis

#### EMG spectral analysis

**Figure 6:**
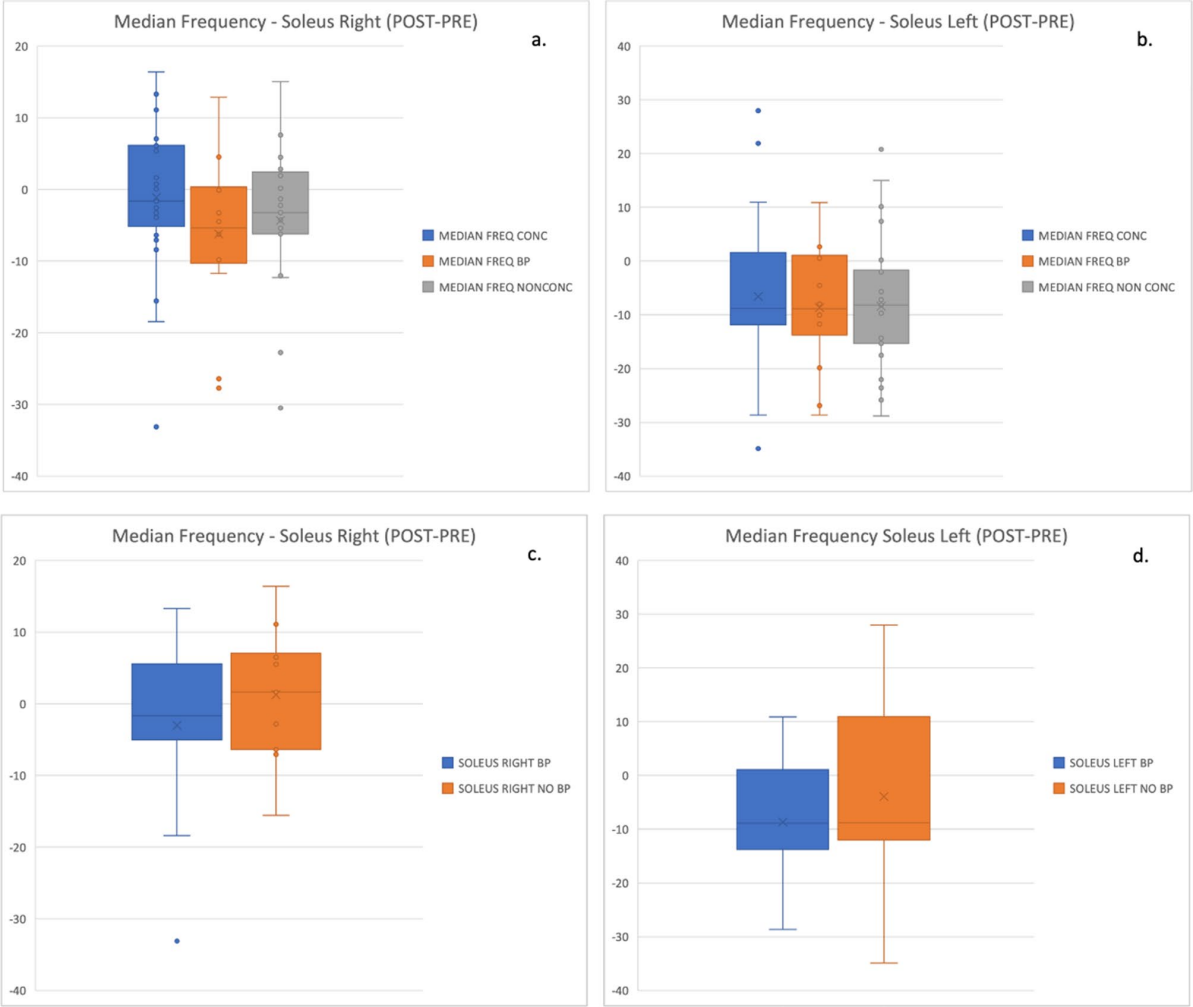
The POST–PRE results for median frequency in the (**a**) right and (**b**) left soleus muscles for the concussion (CONC—blue), concussion with balance problems (CONC BP—orange) and non-concussion groups (NON CONC—grey). Figures (**c**) and (**d**) show the POST–PRE results for median frequency in the left and right soleus muscles for the concussion balance problems vs concussion no balance problems group. When these groups were compared in the POST phase the right soleus showed a significant discrimination between the BP and No BP groups.

Total power, kurtosis, skewness, and median frequency were calculated for each muscle in the concussion and non-concussion groups in both the PRE and POST stages of the experiment. For these POST–PRE results, the muscle that best discriminated the concussion and non-concussion groups was the soleus muscle, for all features except median frequency. A table of the EMG spectral numerical results is shown in the supplemental material (Table S1). Two subgroups from the concussion group were also analyzed, based on their responses to the SCAT5 symptom questionnaire: those experiencing balance problems (BP) and those not experiencing balance problems (No BP). Median frequency results for the right soleus of the BP group showed significance (*p* = 0.0075) in the POST phase of the experiment when compared to the no BP group. The POST–PRE results in the median frequency of concussion, non-concussion and BP groups were not significant. Median frequency results for the soleus are presented in Fig. 6.

#### EMG area analysis

The changes (POST–PRE) in the area under the EMG signal were not significant for any group, nor do the area measurements show any differentiation between the concussion and non-concussion group. (Further information about the EMG area results is available in the supplementary information and Figure S1).

### Heart rate analysis

Heart rate was measured in beats per minute (bpm) PRE and POST for each participant. The non-concussion participants experienced a minor, non-significant, change in heart rate. The concussion group participants experienced a larger change in heart rate from PRE to POST. However, the difference between POST and PRE (POST–PRE) was not significant for the concussion group. (Further information about the HR is available in the supplementary information and Figure S2).

### Classification analysis

**Figure 7:**
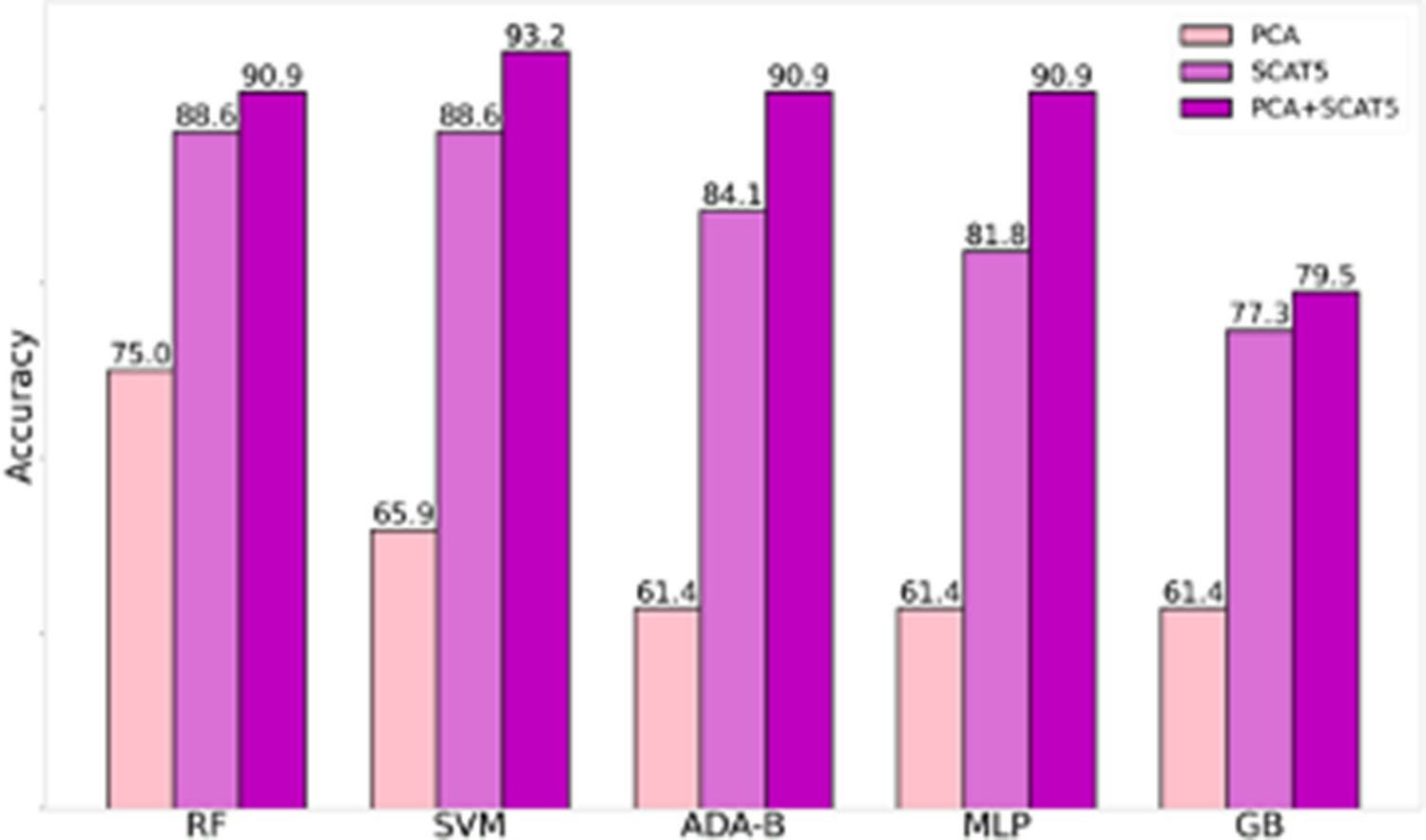
The results of 5 different algorithms (RF- Random Forest, SVM—Support Vector Machine, ADA-B—Adaptive Boosting , MLP- Multilayer Perceptron, GB-Gradient Boosted) and combinations of features. Highest accuracy in all algorithms is achieved using both the PCA and SCAT5 features, but highest accuracy overall (95.5%) when computed with an SVM algorithm.

Three different features selections were used as predictive features to demonstrate the capability to classify concussed and non-concussed athletes through a supervised machine learning approach and a 10 k-fold cross validation. Tree-based, linear-based and simplified artificial neural networks algorithms were used to consider different algorithmic strategies for the classification. Table 4a shows the accuracy, sensitivity and specificity obtained with the 9 PCA features: the highest accuracy of 72.7 was reached with a Random Forest (RF) model. In contrast, the most heightened sensitivity of 57.9 was achieved with Gradient Boosting (GB), another tree-based model. Table 4b shows the results from the SCAT5 features models, with RF and the linear-based Support Vector Machine (SVM) model having the highest accuracy of 88.6 and the simplified artificial neural networks of the Multilayer Perceptron (MLP) model having the highest sensitivity of 89.5. The best results were achieved by merging the two features sets (Table 4c) with all the models exceeding 90 in accuracy except (GB), getting a significant 95.5 with SVM and 93.2 with Ada Boosting (ADA-B) (Fig. 7). All the sensitivity and specificity results for all the models with the combined feature selection are noteworthy. It is also worthy of underlining that the linear model SVM is the one from which the best accuracy is obtained with the 31 features. At the same time, the one based on the artificial neural network MLP gives the best sensitivity of 96.0. Tree-based models work with good results apart from the GB, which, in all cases, is the one with the least significant accuracy results.

**Table 4:** Concussion classification evaluation metrics for the three different feature selections proposed: a. The 9 PCA features; b. the 22 SCAT5 features; c. the 31 combined features of PCA and SCAT5.

Algorithm	Accuracy	Sensitivity	Specificity
**PCA Features (9)**			
RF	72.7	68.4	76.0
SVM	61.4	30.8	92.0
ADA-B	68.2	57.9	76.0
MLP	70.5	63.2	76.0
GB	63.6	73.7	56.0
**SCAT5 Features (22)**			
RF	88.6	84.2	92.0
SVM	88.6	84.2	92.0
ADA-B	84.1	73.7	92.0
MLP	81.8	89.5	76.0
GB	77.3	78.9	76.0
**PCA + SCAT5 Features (31)**			
RF	90.9	89.5	92.0
SVM	95.5	94.7	96.0
ADA-B	93.2	94.7	92.0
MLP	90.9	96.0	86.0
GB	79.5	78.9	80.0

## Discussion

The results of this study support a novel method in concussion assessment by evaluating self-reported concussion symptoms and history against neural and postural responses acquired in a BioVRSea environment, with machine learning used to demonstrate the classification ability of this model. We hypothesized that (1) The Icelandic versions of the SCAT5 symptoms checklist, although not a diagnosis tool, can be used to differentiate between concussed and non-concussed athletes, (2) changes of CoP, heart rate, EMG and EEG data can quantitatively measure concussion and concussion symptoms, and (3) machine learning techniques using SCAT5, and neurophysiological parameters can improve assessment of concussion.

Participants in this study were all female athletes, either with a self-reported history of concussion or no history of concussion. Participants were matched in age and divided almost equally among the two groups. The participants with a history of concussion had a significantly higher score on the concussion symptom scale (SCAT5). The symptoms on the SCAT5 scale are non-specific to concussion, meaning that symptoms can be seen in those with no history of concussion^89^. Nevertheless, the SCAT5 has been recognized as a concussion assessment tool and is used to assess symptoms after a concussion^4^. Only the Icelandic version of the SCAT3 has been validated in an Icelandic sample, with more studies being recommended^132^. The scale was therefore administered to all the athletes to assess if it differentiated between those with and without a history of concussion. Results supported that it did. The most frequently reported symptoms among those with a concussion history were headaches, fatigue and low energy, drowsiness and nervousness, and the symptom reported by the fewest was feeling like in a fog, reported by almost 30%. This is in stark contrast to the non-concussion group where the most frequently reported symptom, headache, was reported by 32% and was the only symptom reported by more than 30%. This was expected as headache is the most common post-concussive symptom, followed by troubles with memory and concentration, dizziness, and balance problems. In addition, results show that the correlation between concussion count (no concussion vs. concussion) and symptoms severity score was significant with concussion history resulting in a higher severity score for concussion symptoms.

To quantify concussion symptoms, changes of CoP, heart rate, EMG and EEG measures were analyzed against reported concussion history and by concussion subgroups represented by specific symptoms reported on the SCAT5. CoP features showed a larger change in six features in the displacement and velocity analysis. Other studies support these results, having previously found that subjects who have experienced concussions show alterations in displacement and velocity CoP beyond the date of clinical recovery or return to play^126^. According to results from a study^128^, the disruption induced in the Postural Control (PC) by a concussion may be observed from the CoP even months after the injury. Since there are many mechanisms involved in maintaining the stability of the body, the stabilogram is a good candidate to evaluate impairments in case of concussions. To evaluate any decrease in the static stability we considered the following: TOTEX, as a decreased instability may lead to an increased movement compared to more stable subjects; RDIST follows a similar rationale, accounting also for the distance from the center as a measure of instability; MVELO^129^ accounts for both spatial and frequency components, and the velocity may have a significant role in anticipatory movement response^130,131^; The Main Axis of the 95% confidence ellipse^106^ indicates the magnitude of the principal component of motion, a greater axis length is associated to a decreased stability; and SD_AP reflects the variability in the Antero-Posterior Signal^129^. We decided to include the Postero_Magnitude feature to assess whether extreme and rare oscillations may be signs of postural control impairments although further research should investigate this phenomenon better. Finally, we investigated the AP and ML signals in the frequency domain computing the Power Spectral Density (PSD) with Fast Fourier Transform (FFT) and used the log–log plot to compute the exponent of the PSD power law. The only statistically significant difference was on AP direction when comparing concussion subgroups related to balance problems, thus the AP direction may have a critical role in maintaining balance compared to ML direction in case of concussion.

The EEG results indicated a significant difference (at some frequency bands) between those with a history of concussion and those with no history of concussion when participants had to maintain postural control and balance. Activity on the theta band was increased in those with a history of concussion. The theta wave activity has been associated with the ability to encode new information, and is correlated with cognitive performance, memory in particular^59,66^, and may appear normal during relaxed wakefulness^60^. Theta activity has been observed to increase during cognitive tasks compared to motor tasks, indicating an active role in problem solving^61^ and has been associated with tasks that need more attention and cognitive demands^62^. An increase in theta among those with a history of concussion when compared to those with no history of concussion may indicate a need for more attention and cognitive effort than those who do not have a history of concussion. The increase in theta was additionally present in concussion subgroups among participants that reported having difficulty concentrating, feeling pressure in the head, and feeling fatigue and low energy. Further supporting their reports of concussion symptoms and offering an objective way to assess post-concussive symptoms. Concussion history has been connected to cognitive impairment^22,63^, like attention and reaction^64,65^, and cognitive fatigue has been suggested as a sub-type of concussion^19^. The increase in theta may support this type of cognitive impairment and indicates that the task was more demanding for those with a history of concussion. It is worth mentioning that theta increase has also been found in the frontal and central regions for postural control adaptation and habituation^87^, highlighting its activation during balance and postural control disturbance^88^.

EMG area measures showed differences between the two groups based on signals from the tibialis anterior (TA) when participants had to maintain postural control in the PRE and POST stages. The calf muscles are active agonists in upright posture^18^, driving the main action of maintaining upright balance. Those with a history of concussion showed a trend towards higher muscle activation in the TA when maintaining balance POST, although the difference was not significant. This could indicate postural control and balance problems, resulting in a more active muscle when trying to stabilize in an unstable environment. Impaired postural control has been connected to concussion history^21^, as well as impairment in visual and vestibular systems^4,20,51^. Concerning the EMG spectral analysis, all the features computed, namely Total Power, Kurtosis, Skewness, and Median Frequency, showed a stronger statistically significant difference between PRE and POST in the concussion group compared to the non-concussion group. Among the muscles analyzed, the soleus showed the maximum discriminative power to differentiate between the two groups and a coherence between the results obtained from right and left sides. Indeed, for the concussion group, all the features, except the median frequency, exhibited a strong, statistically significant difference between the PRE and POST while non statistically significant differences were found in the same features for the non-concussion group. For both groups, no difference in median frequency was found, which implies that the task did not lead to muscle fatigue. In the concussion group a more symmetrical, Gaussian-like, spectrum shape was observed in the POST compared to the PRE, as indeed kurtosis and skewness showed statistically lower values. Finally, in the concussion group a statistically higher Total Power was found in the POST compared to the PRE suggesting an increase in the muscle contraction force. The further statistical analysis, aimed to compare within the concussion group subjects who experienced balance problems versus subjects who did not experience balance problems, showed once again the significant discriminative power of the right soleus muscle. In fact, in this analysis, contrary to the previous one, the median frequency feature has proved to be the most significant feature being able to discriminate concussed subjects (of which are 92% right-handed) with and without balance problems in the post phase. We can conclude that the median frequency features together with the other spectral features – namely Total Power, Kurtosis and Skewness—could be potential biomarkers to predict and explain concussion and its related symptoms (e.g., balance problems).

Output from heart rate (HR) sensors showed that those with a history of concussion had a higher HR than those with no history of concussion when postural control and balance were also considered. The HR variance was higher for both groups before the balancing/postural control task began in the VR. HR was higher for both groups at the start of the task and may be due to stress regarding the unknown task at hand. However, despite the HR being higher for both groups, the variance was higher for those with a history of concussion. On average, the HR was high in the hands-off part of the VR task and lowered when participants could hold on to the handle in front of them. HR measures have shown potential as an additional measure to potentially index the ANS in reaction to concussion^47–49^. These results support the possibility that HR rate measures could give added information when mapping out physical markers of concussion.

ML results are promising and demonstrate that concussion can be assessed by the biometric measurements from BioVRSea and SCAT5, especially if combined. The use of only neurophysiological measures allows a decent classification accuracy of almost 73% with RF, which demonstrate the ability of the BioVRSea multi-biometric system not only to evaluate sick and not motion sick people as previously published^75^ but also to individuate concussed and not concussed athletes. These results increase if the BioVRSea features are combined with the SCAT5 features. The latter alone can produce an 88.6% accuracy, which is remarkable but not innovative. The novelty of the results is the successful combination of measured and self-reported parameters as seen in Table 4c, with an accuracy of 95.5%. Furthermore, the combination of these two different measurement approaches provides a novel tool that can be implemented also for monitoring effect of treatment, to develop rehabilitation strategies, or even to support insurance assessment.

Discussing the different algorithmic approaches, we can state that the linear system works better when the SCAT5 features are involved. In^104^, SVM was also used for a similar purpose with a larger but unbalanced dataset. Our dataset is smaller and includes fewer features, but they are quicker to assess, and the balanced number of concussed and not-concussed subjects strengthens the obtained classification accuracy. SVM was also successfully performed in^105^ for an individual-level concussion detection starting from only EEG features: this can suggest that as a probable future development of the ML concussion analysis, a focus on more specific EEG features can be performed to understand better how the BioVRSea system can eventually mitigate or improve concussion influence on the brain. The tree-based algorithmic models confirm that the BioVRSea multimetric measurements can be better processed with this approach, like in^75^. The simplified neural network MLP suggests us that a more complicated neural network model can be, with a larger population, worth trying to increase the classification ability of the model.

## Strengths and limitations

This study has several important strengths, as well as several limitations. It is challenging to confirm concussions, especially when carrying out a retrospective study. Using medical records to confirm concussion is the gold standard^15^. However, high-risk groups like athletes do not always seek out medical assistance^16,58^. Medical records could therefore be inaccurate and could result in misclassification of a participants´ concussion history. In this study, participants were asked how many concussions they had received after being given a concussion definition. This increases the reliability of self-reporting^58,67,85^. A strength is that new technological advances were used to assess concussion symptoms, finding quantitative patterns related to brain, muscles, and CoP which were able to discriminate conditions such as balance problems, difficulty concentrating, pressure in the head, and fatigue/low energy. VR technology was used to provide a secure environment where responses to a postural control task was studied. Finally, the main strength of this work is the multi-faceted approach to assess concussion as recommended in the scientific literature^24^.

One limitation to this study is that participants were not asked about prior balancing problems like inner ear problems before they participated. As Manley^22^ pointed out there are several methodological limitations in concussion research; one is that most studies do not include the possibility of a third variable affecting the outcome, e.g., substance use, genetics, family history, and mental and cognitive health^22^. In this study, both groups were athletes; some retired and others active, they are all females, and mean age and level of education are similar. Groups are, therefore, well matched in other respects.

As participants were all actively or historically involved in contact sports and as such this is a group at elevated risk for receiving sub-concussive injuries throughout their careers^50,70^. The comparison between the concussion and non-concussion groups is not a comparison between a concussion group and a normal population. Both groups will likely have received repeated head and body impacts, with possible sub-concussive blows. Sub-concussive blows can result in concussion symptoms, including problems with balance^133,134^ or the neck^68^, but neck proprioception is extremely important in postural control. Neck injury not related to a concussion is also a possibility in this groups and should be considered in future studies. Having all participants possibly exposed to injuries affecting postural control and dizziness is both a strength and a limitation. It’s a limitations as some participants in the non-concussion group could belong to concussion group, the only clear distinction when placing participants in groups for analysis is their report of concussion history. On the one hand, because the groups are so similarly matched this type of injuries are controlled for. Measures evaluated in this paper could therefore be differentiating between the concussed vs non-concussed.

Hormonal influences were also not studied in this experiment. There is robust evidence in animal models, that higher estrogen and progesterone levels at time of injury can have a neuroprotective effect in the female animal, particularly in the proestrus phase of the cycle^69^.

The limited number of participants limits the ML algorithm’s predictive capabilities, but the balanced number of concussed and not-concussed subject is a strength. Recruitment of more participants in the future could improve the results in terms of accuracy, and more complicated algorithmic models can be implemented.

## Conclusion

In this study we developed a novel paradigm to measure postural control. We demonstrated that we can discriminate between concussion and non-concussion groups using the BioVRSea setup and particularly, symptoms associated with concussion, especially with balance problems, follow a pattern that can be quantified. This study shows the value of a subject-specific multi-faceted postural control assessment.

## Methods

### Participants

Participants were all female athletes (*N* = 54), competing at the highest level in Iceland in basketball (16.7%), handball (35.2%), soccer (38.9%), ice hockey (5.6%), or martial arts (3.7%). Mean age was 38.4 (SD = 7.7). Almost half of the participants had a history of concussion/s, 48.1% (*n* = 26) half had no concussion history, 51.9% (*n* = 28). Mean years since retirement was 4.3 years (SD = 4.9). All participants had a college or a university diploma, 48% had an MA or an MS degree. Athlete profiles divided by groups can be seen in Table 5.

**Table 5:** Age and athletic status of participants.

	Athletes with a history of concussion (*N* = 26)	Athletes with no history of concussion (*N* = 28)
Age mean (SD)	30.5 (6.9)	29.8 (8.2)
Retired % (*n*)	69.2 (18)	57.1 (16) *
Active % (*n*)	30.8 (8)	39.3 (11)

*One athlete without a history of concussion did not clarify if she was retired or still active (3.6%).

### Procedure

The athletes were first contacted via snowball sampling using social media pages in 2018 when the athletes participated in an online study, during which they answered questions about concussion history and indicated whether they had sustained a concussion or not after being provided with a concussion definition^67^. In 2019, participants were contacted again using social media and asked if they wanted to participate in a concussion study where biometric measurements would be made while they were in a virtual reality (VR) environment.

### Experimental procedure

#### Written information and informed consent

All participants were provided written information about the study prior to signing an informed consent document. The study protocol was approved by the Icelandic National Bioethics Committee (no: 17–183-S1).

#### Concussion definition

Participants were read a concussion definition and were asked if they had sustained a concussion. The definition was based on the Berlin Consensus statement on concussion in sport from 2016^4,67,67,127^.

#### SCAT5 questionnaire

All participants completed the symptoms scale from The Sport Concussion Assessment Tool 5 (SCAT5)^13,26,67^ before the experiment. The overall score is calculated by the sum of each participant’s responses. The SCAT5 has been recognized as a concussion assessment tool^4^.However, the SCAT5 should not be used as a diagnostic tool but as part of an assessment battery^26^. The SCAT5 symptoms checklist has not been validated in a Icelandic sample only the SCAT3^132^. The scale has 22 items, each item scoring from 0 to 6, indicating the severity of the symptom^26^. In this study, it was hypothesized that the Icelandic versions of the SCAT5 symptoms checklist, could be used to differentiate between concussed and non-concussed athletes, it was used to validate the self-reported concussion status and assess for each group the changes of some physiological conditions associated with our experiment.

#### Virtual reality experiment

The participants were then prepared for the virtual reality and physiological measurement part of the experiment. This involved the placement of a wet 64-electrode EEG cap (Sampling frequency 4096 Hz, ANTNeuro, Hengelo, The Netherlands), six wireless EMG sensors (Sampling frequency 1600 Hz Kiso ehf., Reykjavík, Iceland) on the tibialis anterior (TA), gastrocnemius lateral (GL), and soleus (S) muscles of each leg, and a heart rate sensor (sampling frequency 1 Hz, PolarBeat, Kempele, Finland) strapped around the chest. The EEG amplifier (ANTNEuro, Hengelo, The Netherlands) was connected to the cap and placed in a backpack with a tablet used for EEG signal acquisition. The participant put on the backpack and was instructed to step onto the force plates after removing their shoes. The position of the feet was in bipedal stance with feet hip width apart, while standing on the force sensors (Sampling frequency 90 Hz, Virtualis, Clapiers, France). The force sensors on the moveable platform (Virtualis, Clapiers, France). Finally, the participant dons the VR goggles.

The experimental protocol was then explained to the participant. The explanation included that they should stand quietly on the platform with their hands by their side observing a mountain view for the first 2 min of the experiment. Then, the scene in the VR goggles would change, beginning the sea simulation. The participants were instructed to remain standing quietly with their hands by their side for the first 35 s of the sea simulation. There was no platform movement in this part of the experiment, and it is called the PRE phase of the experiment. After 35 s of quiet standing watching the sea simulation, the participant was instructed to hold onto the bars in front of them. The platform then began synchronized movement with the sea scene in the VR goggles, with 25%, 50% and 75% of maximal wave amplitude. In this central part, each segment lasted 40 s and the participant held the bars of the platform while continuing to observe the sea simulation. Finally, the platform stopped moving and the participant was asked to remove their hands from the bars and attempt to stand quietly with their hands by their side for the final 40 s of the experiment. The sea scene was still observed by the participant for the final 40 s. This is called the POST phase of the experiment; it is performed identically to the PRE phase but after the participant has performed movement in the central part of the procedure. A table of the VR experiment protocol is shown below in Table 6, Fig. 1 shows a schematic of the experimental setup. Each participant took part in a single trial according to the experimental protocol.

**Table 6:** Virtual Reality/Platform Synchronization Protocol.

Time (s)	Segment	VR Scene	Position of hands	Platform
0–120	Baseline	Mountains	By side	Stationary
120–160	PRE	Sea	By side	Stationary
160–200	25%	Sea	On bars	Moving
200–240	50%	Sea	On bars	Moving
240–280	75%	Sea	On bars	Moving
280–320	POST	Sea	By side	Stationary

The operator can set the frequency of the waves between 0.5 Hz and 3 Hz and the amplitude of the waves between 0 and 2. During the simulation, we vary the amplitude of the platform movements from 0% up to 75% of the platform’s maximal amplitude. Two different protocols were used at random throughout the study. The ‘soft’ protocol was defined as a wave frequency of 1 Hz with an amplitude of 0.6 while the ‘hard’ frequency was defined as a wave frequency of 3 Hz with an amplitude of 0.5. Each participant experienced either the hard or soft protocol once while taking part in the experiment.

Randomly selected amplitude of the experiment (soft or hard protocol) is made to mimic the variety of “sea behavior,” to cover a wide possibility to trigger a postural control response.

#### Data acquisition

During each protocol, muscle, brain, heart, and CoP data were acquired using the following technologies:

- Brain electrical activity was measured using a 64-channel wet electrode cap (sampling frequency of 4096 Hz) from AntNeuro, Hengelo, the Netherlands.
- Muscle electrical activities from the lower limbs was acquired using six wireless EMG sensors (sampling frequency of 1600 Hz) placed on the tibialis anterior (TA), gastrocnemius lateral (GL), and soleus (S) muscles of each leg (Kiso ehf, Reykjavik, Iceland).
- Heart rate was measured using a chest heart sensor (Polar Electro, Kempele, Finland, sampling frequency 1000 Hz).
- Force Plate measurements were made using 4 sensors located under each foot platform. The sensors give information about the center of mass in the Antero-posterior and Medio-Lateral axis (Virtualis, Clapiers, France, sampling frequency 90 Hz).

#### Post processing and calculations

The data from each measurement were divided into 6 segments, corresponding to each stage of the protocol. Data for the EEG, EMG and CoP were analyzed by calculating POST–PRE (POST minus PRE) paradigm.

##### Division into subgroups by symptoms

Subjects from the concussion group were further divided into subgroups for quantification of some symptoms from the SCAT5 questionnaire.

For instance, CoP measures may be different among the concussion participants that reported balance problems, compared to those that did not/non-concussion group. The following symptom-based subgroups were formed from the concussion participants for the following measurements:

For EEG: Pressure in the head symptom group (17 subjects), Fatigue/Low Energy symptom group (19 subjects), difficulty concentrating symptom group (16 subjects).

For HR: Nervous/Anxious group (20 subjects), Fatigue/Low Energy Group (19 subjects).

For EMG: Balance problem symptoms group (14 subjects).

For CoP: Balance problem symptom group (14 subjects).

##### EEG

The EEG analysis was performed on the spectral domain. The focus was put on the difference between the PRE and POST segments. The considerable electrodes were identified between those two segments.

The EEG was recorded using a 64-electrode channel system with an electrooculogram (EOG) electrode placed below the right eye and a ground electrode placed on the left side of the neck. Data pre-processing and analysis were performed with Brainstorm^71^ and Matlab_2020b,^72^ using the Automagic toolbox^73^.

For each segment, we removed the 5 first and 5 last seconds, to ensure the data quality and to avoid artefacts. The data were resampled to 1024 Hz. Automagic was used to automatically pre-process every dataset, with a manual inspection at the end. The ICA MARA algorithm was used, with a variance of 20%. The data were notch filtered at 50 Hz. A high pass and low pass filter were set respectively to 1 Hz and 45 Hz. Finally, the bad electrodes were interpolated. Each segment was on average interpolated up to 10%.

The power spectral density (PSD) was computed for each epoch with ‘Welch’s method, with the following frequency bands: delta (1–4 Hz), theta (4–8 Hz), alpha (8–13 Hz), beta (13–30 Hz), low gamma (30–45 Hz). The relative power of each band was then computed obtaining a total of 5 EEG-related features.

##### EMG

Two types of EMG analysis were performed on the participants data. The area under the rectified and smoothed EMG curve was calculated for each electrode, as well as spectral analysis of each signal.

EMG data processing was performed using Matlab_2020a^72^. The EMG signal was filtered with a 50th order FIR bandpass filter with cut-off frequencies at 40 and 500 Hz. The integral of the rectified EMG signal for each leg was calculated, resulting in 1 EMG area measurement per muscle for each segment of the acquisition. The area under the EMG curve indicates the excitability and neural drive to the muscle, preceding muscle contraction^57^.

If the two phases, namely PRE and POST, last 40 s we considered a central 30-s time window for each of the two phases to exclude the effects of the transition between phases. Successively, we performed in these time windows an electromyography (EMG) spectral analysis through the analysis of the signal power distribution calculated by the Fast Fourier Transformation (FFT) algorithm and therefore computing the total power spectrum of the EMG signal. For each of the two resulting total power spectra, one for the PRE and the other one for the POST, four frequency-domain parameters were extracted: Total Power, kurtosis, skewness, and median frequency.

The total power is the integral under the spectrum curve which increases with the force of contraction^90^. The kurtosis is a measure of how outlier-prone a distribution is; we used a kurtosis function that considers the following assumptions: the kurtosis of the normal distribution is 3 and the kurtosis is biased. Skewness is a measure of the data's asymmetry around the sample mean; we used a skewness function that considered a biased version. Kurtosis and skewness were chosen because they allow evaluation of any changes in the EMG spectrum. Studies show their valuable contribution in the EMG analysis^91–93^. The median frequency is the frequency that divides the total power area into two equal parts. The median frequency was chosen considering its usefulness in describing fatigue changes^94,95^, including the soleus and gastrocnemius muscles^96^. The analysis of median frequency has also been useful in diagnosis of various disorders. EMG analysis and feature extraction were carried out through MATLAB R2020a.

##### Heart rate

The heart rate data were averaged for the 40 s PRE and POST stages and the standard deviation computed.

##### CoP

**Figure 8:**
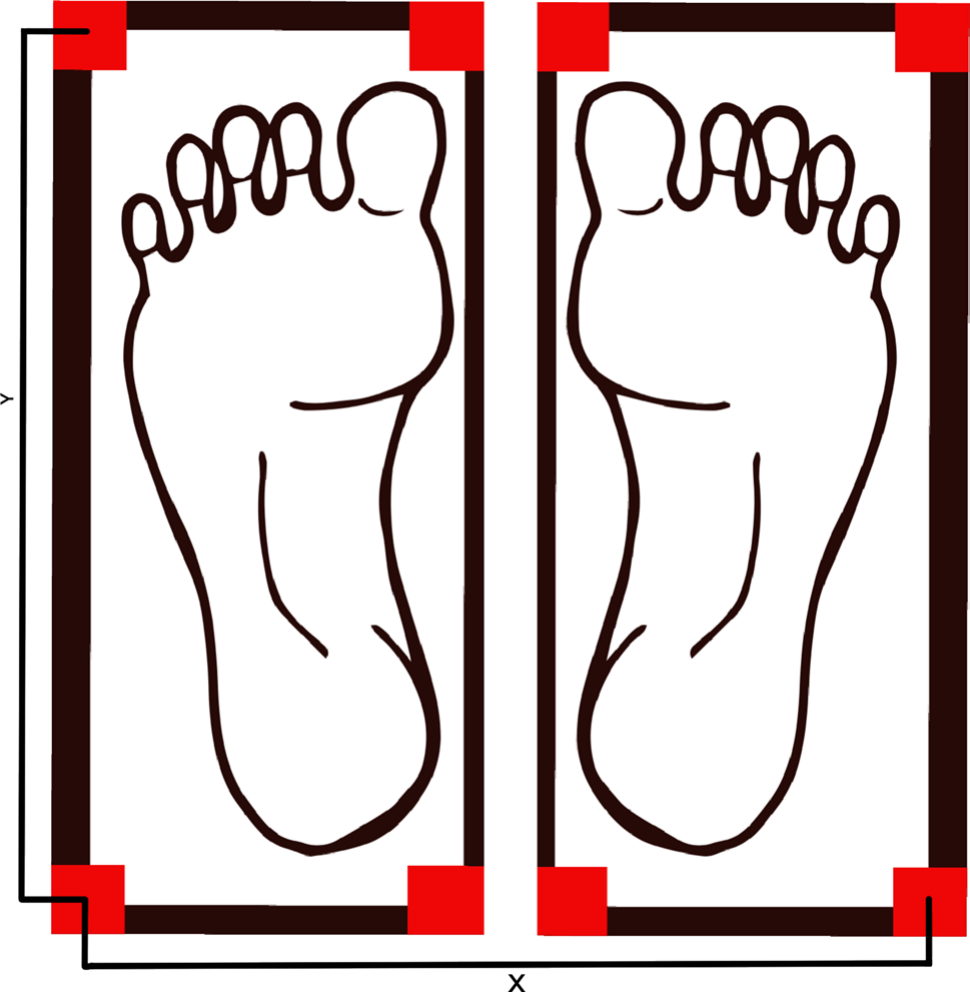
This figure shows the position of the feet as they are placed on the force platform during the experiment. The anterior–posterior displacement is calculated using the formula: ${\text{AP - Displacement}}\left( {{\text{cm}}} \right) = Y\left( {{\text{cm}}} \right) * {\text{StaticVR}}\_{\text{AP/}}2$. Where StaticVR AP is the percentage of the vertical size of the board between −1 and 1. The medio-lateral displacement is calculated using the formula: ${\text{ML\_Displacement }}\left( {{\text{cm}}} \right) \, = \, X\left( {{\text{cm}}} \right)*{\text{StaticVR}}\_{\text{ML}}/2$ Where StaticVR ML is the percentage of the horizontal size of the board between −1 and 1.

Figure 8 shows a schematic of the foot position on the force plate and the formula for calculating the anterior–posterior (AP) and medio-lateral (MLa) displacement is as follows:

Where StaticVR MLa is the percentage of the horizontal size of the board between − 1 and 1.

The processing of the CoP data was performed using Matlab_2021b^72^. During the experiment, the force platform records the movement of the Centre of Pressure (CoP), a projection of the center of mass of the subject on the plane of the machine, also called stabilogram. The CoP data was filtered with a Savitsky-Golay filter with window size 7.

We extract several parameters from the stabilogram for evaluating the postural control response of the subject before (PRE) and after (POST) the perturbation caused by the movement of the platform (25%, 50%, 75%) and the visual cues provided by the VR goggles.

Since during PRE and POST the platform is not moving, the subject is experiencing only the visual cues provided by the movement of the waves, in other words we are evaluating how the postural control changes the movement of the COP and if certain conditions can affect this complex biological system.

The most important geometrical features extracted are the 95% confidence ellipse (axes length, orientation, and area), the mean root square distance from the center (RDIST)^106^, the total excursion (TOTEX) and the position of extreme points (Anterior, Posterior, Left, Right). TOTEX is computed on support plane, on the Antero-Posterior Axis (AP) and Medio-Lateral Axis (ML).

In addition to geometrical feature, we computed the standard deviation and sample entropy^108^ of ML and AP signals and heading change^107^. Following^109,110^ AP and ML Complexity Index (CI) and Multivariate CI are obtained from the multiscale entropy with time scales 1 to 6.

Moreover, we consider the difference between consecutive points to evaluate the Mean Velocity (MV) on the support plane and on each individual axis.

Finally, to understand any link between the chosen set of parameters and the aim of this study we considered the POST features minus the PRE features, and we performed a Wilcoxon signed-rank test to assess the relationship between features and subgroups under analysis. We also tested for statistically significant differences between PRE and POST for each individual variable.

**Figure 9:**
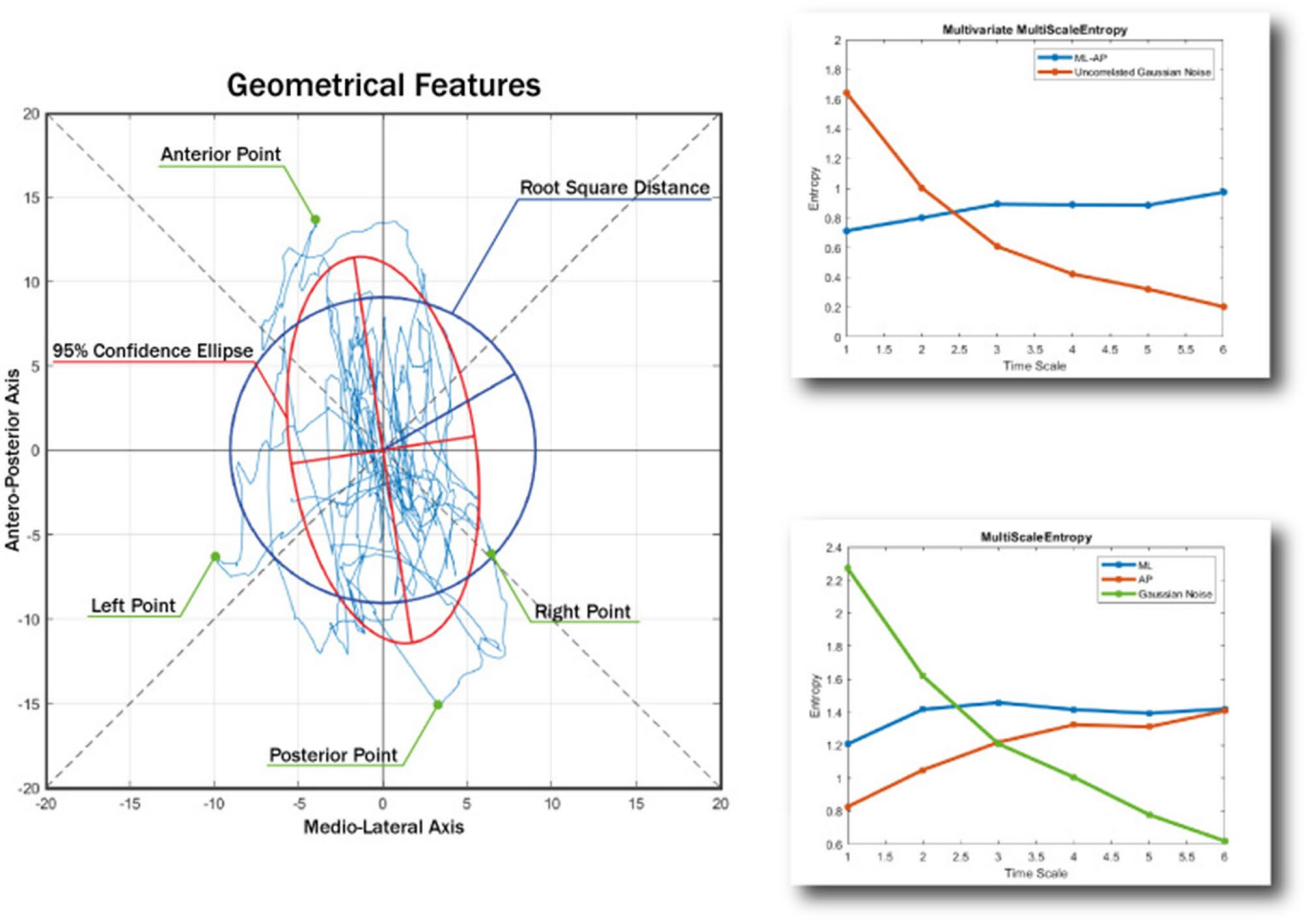
A graphical description of the features used in the CoP analysis. A number of geometrical and spectral features were calculated for the PRE and POST stages of the experiment. The figure shows an example of a stabilogram on the left and how some features are extracted. Similarly, an example calculation of entropy features is shown on the right to give some indication of what was calculated for the entropy measures using the stabilogram data.

A schematic of the force platform features computed is shown in Fig. 9.

##### Statistical tests

###### SCAT5

An independent t-test was used to compare means between concussion and non-concussion groups. Significance level was set at *α* = 0.05.

The hypothesis being tested in all measured quantities was that the POST measurements were significantly different to the PRE.

###### EEG

The statistical significance for each electrode was computed (*t*-test, *α* = 0.05) for the results from the difference Hands-off Post minus Hands-off Pre segments, for each frequency band, within each group (concussed and non-concussed). It was then corrected for multiple comparisons using the False Discovery Rate (FDR) (Benjamini-Hochberg) method^86^.

###### EMG area/heart rate/CoP parameters

The statistical significance for these data sets was computed between the PRE and POST responses via the two-tailed Wilcoxon matched pairs signed rank test with significance set at *α* = 0.05.

###### EMG spectral analysis

We performed a statistical analysis to assess any significant difference between the PRE and POST phases for each muscle frequency-domain parameter and for each group, namely concussion and non-concussion. To achieve this, we performed a non-parametric statistic test for paired samples considering the low sample size (< 30) and based on the result of the normality Shapiro–Wilk test. A two-tailed Wilcoxon matched-pairs signed rank test with a 95% confidence level was considered (definition of statistical significance: *p*-value < 0.05; *p-value < 0.05, **p-value < 0.01). For comparing the POST phases of the balance problem/no balance problem groups, a Mann–Whitney test was used (*p* < 0.05). All statistical tests were performed using GraphPad Prism 9.0.0 software.

### Machine learning analysis—predictive/classification analysis

**Figure 10:**
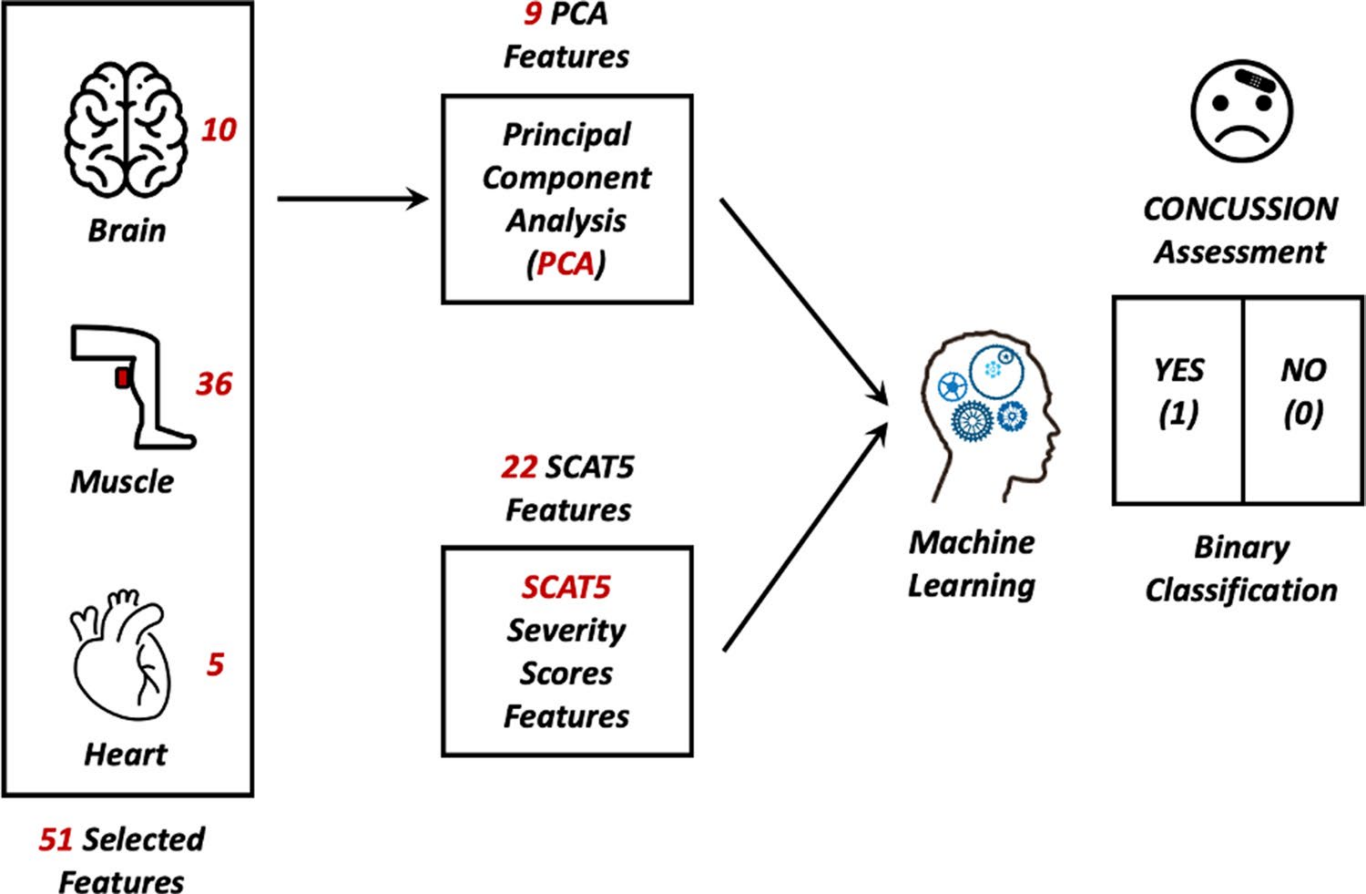
Feature Selection and Machine Learning Workflow. 51 features are selected from Brain, Muscles and Heart signal and on them, the PCA is performed to obtain 9 PCA features used to the binary classification of the concussion assessment using different ML algorithms; 22 SCAT5 features are then used as well as features for the concussion classification; the 22 SCAT5 and 9 PCA are then combined and used together to classify concussed and non-concussed subjects.

The Machine Learning analysis was performed using the popular software named Knime Analytics Platform (v. 4.2.0) to binary classify the participants who self-reported concussion and those who had not (Fig. 10).

The feature extracted from the BioVRSea measurement provides a total of 51 features. (Table 7). These are the 5 HR average for each experiment phase, the 10 EEG features (Delta, Theta, Alpha, Beta and Low Gamma for the PRE and POST phase) the 24 EMG features (PRE and POST areas for each of the six muscles sensors and the PRE and POST median frequencies in each muscle) and the 6 more significant CoP features (see Fig. 3 and Table 7). On these selected 51 features, the Principal Component Analysis (PCA)^74^ is performed: this algorithm generates 9 PCA features used as input for the binary classification of the concussion participants (Fig. 10). The 22 Severity Score Features, SCAT5 (see Fig. 2), are also used for the concussion classification. Finally, the 22 SCAT5 and the 9 PCA are combined, obtaining in total three different feature selections as input to the binary classification ML models:

1. 9 PCA features from brain, muscles, heart, and CoP signals from the *BioVRSea* measurements
2. 22 SCAT5 features from the concussion questionnaire
3. 31 PCA + SCAT5 features

**Table 7:** The 51 selected features from the BioVRSea measurements.

Signal	Experiment phase	Description	Total number
HR	Pre	Average heart rate	5
	25		
	50		
	75		
	Post		
EMG	Pre	6 Muscles Areas	12
	Post		
	Pre	6 Muscles Median Frequency	12
	Post		
EEG	Pre	Alpha–Beta–Theta–Delta–Low-Gamma	10
	Post		
CoP	Pre	TOTEX–MDIST_AP- MVELO–Ellipse_Main_Axis – SD_AP- Postero_Magnitude	12

The algorithms implemented are the same previously considered in^75^: they are Random Forest (RF)^76^, Gradient Boosting (GB)^79^, Ada-Boosting (ADA-B)^80^, Support Vector Machine (SVM) ^81^ and Multilayer Perceptron (MLP)^82^. These algorithms have been chosen to consider as many different approaches as possible. RF, GB, and ADA-B consider a tree-based approach, which is demonstrated to be very efficient in the biomedical engineering literature^77,78^; SVM uses a linear system while MLP is a class of artificial neural networks. Considering these three different algorithmic approaches, we can have a complete vision of the three feature selections' ability to classify self-reported concussion status with higher or lower accuracy.

The evaluation metrics used to assess the performances of the algorithms into the classification's tasks are the standard accuracy, sensitivity, and specificity ^83^. These metrics were computed using the K-Fold Cross Validation with ten different seeds ^84^.

## Ethics Statement

The study was approved by the National Bioethics Committee (no: 17–183-S1 and VSN-20–101). The study followed all relevant guidelines for the study. The participants provided written informed consent.

## Supplementary Information

Supplementary Information.
