# Supplementary material for: Towards defining biomarkers to evaluate concussions using virtual reality and a moving platform (BioVRSea)
Source: Sci Rep. 2022 May 30;12:8996. doi: 10.1038/s41598-022-12822-0 (PMC9151646; doi:10.1038/s41598-022-12822-0)
Supplement: Supplementary file 1 — Supplementary Information. [file 41598_2022_12822_MOESM1_ESM.docx]

**EMG Spectra Numerical Results**


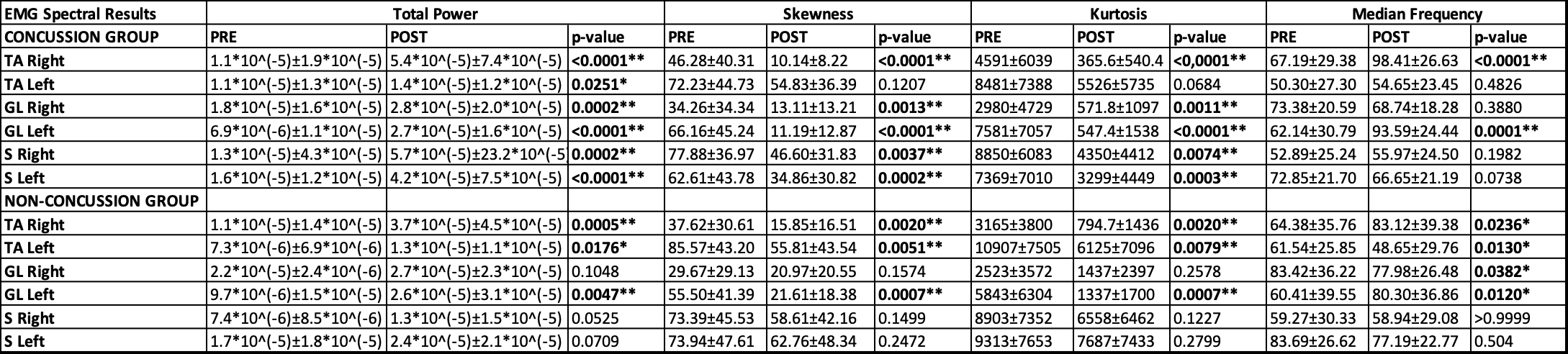


* - p < 0.05

** - p < 0.01

**Table S1 – Numerical results for EMG Spectra.**

Thi stable shows the POST-PRE results for four spectral features (Total Power, Skewness, Kurtosis, and median frequency) per each muscle measured in the experiment (Tibialis Anterior, Gastrocnnemius Lateral and Soleus Left and Right). Bold values are statistically significant.

**EMG Area**

The changes in the area were not significant for any group, nor do the area measurements show any indication of differentiating between the concussion and non-concussion group, with the possible exception of tibialis anterior on the left and the soleus muscles. These muscles appear to have a slightly elevated mean difference in the concussion group than the non-concussion group, possibly indicating a greater excitability in the POST stage of the experiment.

**A)**


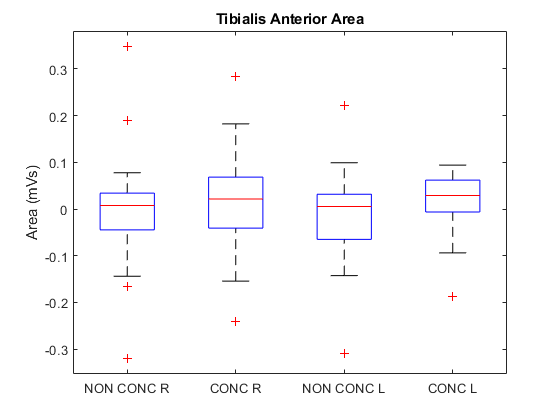


**B)**


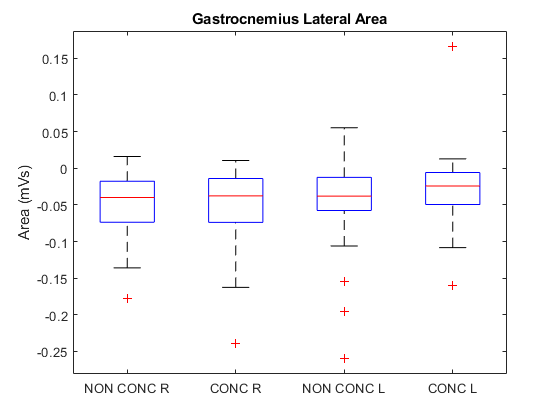


**C)**


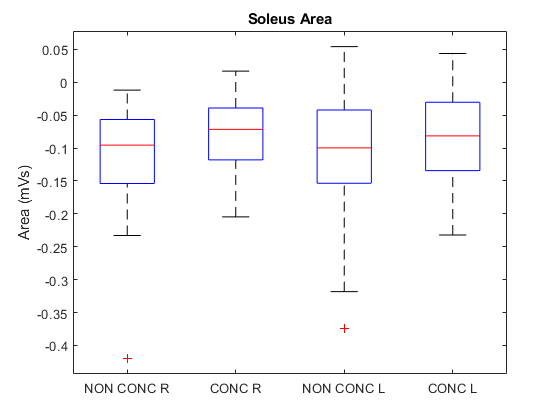


**Supplementary Figure S1 – EMG Area Measurements.** Supplementary Figure S1 shows the difference between the PRE and POST stages (PRE measurement subtracted from POST, POST-PRE) for EMG area. NON CONC R or L represents the non-concussion group and indicates the right (R) or left (L) leg. Similarly, CONC R or L represents the concussion group.

1. This figure shows the difference in the area (POST-PRE) for the left and right tibialis anterior of both the concussion and non-concussion groups.
2. This figure shows the difference in the area (POST-PRE) for the left and right gastrocnemius lateral of both the concussion and non-concussion groups
3. This figure shows the difference in the area (POST-PRE) for the left and right soleus of both the concussion and non-concussion groups.

The changes in the area were not significant for any group, nor do the area measurements show any indication of differentiating between the concussion and non-concussion group, with the possible exception of tibialis anterior on the left and the soleus muscles. These muscles appear to have a slightly elevated mean difference in the concussion group than the non-concussion group, indicating a greater excitability in the POST stage of the experiment.

**HR Results**

Heart rate results we analyzed POST-PRE for concussion and non-concussion groups. No results were significant. A number of symptom subgroups from the concussion group were also analyzed: fatigue/low energy, nervous/anxious, and more emotional. No significant changes were found between the PRE and the POST stage. A graph showing the evolution of the HR throught the whole experiment is shown in Figure S2. The concussion and non-concussion groups are clearly distinguished, although the POST-PRE does not distinguish them. The POST-PRE for the concussion and non-concussion groups is also shown, the change from POST to PRE does not discriminate the groups.


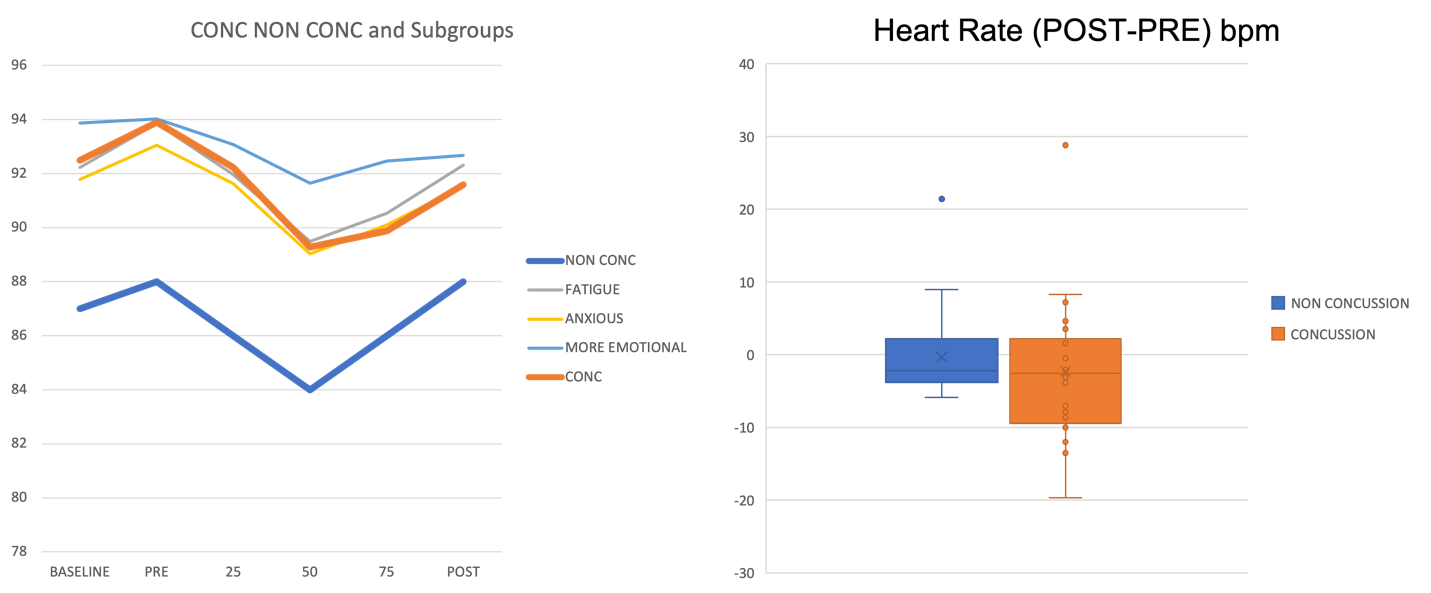


**Figure S2 – Evolution of Heart Rate throughout the experiment and POST-PRE results for Concussion and Non-concussion Groups**
